# Possible Role of Different Yeast and Plant Lysophospholipid:Acyl-CoA Acyltransferases (LPLATs) in Acyl Remodelling of Phospholipids

**DOI:** 10.1007/s11745-015-4102-0

**Published:** 2015-12-07

**Authors:** Katarzyna Jasieniecka-Gazarkiewicz, Kamil Demski, Ida Lager, Sten Stymne, Antoni Banaś

**Affiliations:** Intercollegiate Faculty of Biotechnology, University of Gdańsk and Medical University of Gdańsk, Kładki 24, 80-822 Gdańsk, Poland; Department of Plant Breeding, Swedish University of Agricultural Sciences, 230 53 Alnarp, Sweden

**Keywords:** LPCAT, LPEAT, Ale1, Slc1, Microsomal preparation, Phospholipids

## Abstract

Recent results have suggested that plant lysophosphatidylcholine:acyl-coenzyme A acyltransferases (LPCATs) can operate in reverse in vivo and thereby catalyse an acyl exchange between the acyl-coenzyme A (CoA) pool and the phosphatidylcholine. We have investigated the abilities of Arabidopsis AtLPCAT2, Arabidopsis lysophosphatidylethanolamine acyltransferase (LPEAT2), *S. cerevisiae* lysophospholipid acyltransferase (Ale1) and *S. cerevisiae* lysophosphatidic acid acyltransferase (SLC1) to acylate lysoPtdCho, lysoPtdEtn and lysoPtdOH and act reversibly on the products of the acylation; the PtdCho, PtdEtn and PtdOH. The tested LPLATs were expressed in an *S. cervisiaeale1* strain and enzyme activities were assessed in assays using microsomal preparations of the different transformants. The results show that, despite high activity towards lysoPtdCho, lysoPtdEtn and lysoPtdOH by the ALE1, its capacities to operate reversibly on the products of the acylation were very low. Slc1 readily acylated lysoPtdOH, lysoPtdCho and lysoPtdEtn but showed no reversibility towards PtdCho, very little reversibility towards PtdEtn and very high reversibility towards PtdOH. LPEAT2 showed the highest levels of reversibility towards PtdCho and PtdEtn of all LPLATs tested but low ability to operate reversibly on PtdOH. AtLPCAT2 showed good reversible activity towards PtdCho and PtdEtn and very low reversibility towards PtdOH. Thus, it appears that some of the LPLATs have developed properties that, to a much higher degree than other LPLATs, promote the reverse reaction during the same assay conditions and with the same phospholipid. The results also show that the capacity of reversibility can be specific for a particular phospholipid, albeit the lysophospholipid derivatives of other phospholipids serve as good acyl acceptors for the forward reaction of the enzyme.

**Electronic supplementary material:**

The online version of this article (doi:10.1007/s11745-015-4102-0) contains supplementary material, which is available to authorized users.

## Introduction

Lysophospholipid:acyl-CoA acyltransferases (LPLATs) are a ubiquitous group of enzymes that can be found in animals, plants and fungi [1–6]. They can utilise a broad spectrum of both lysophospholipids and acyl-CoAs, producing different types of phospholipids but substrate specificity of different LPLATs differs substantially. In yeast, two types of LPLATs have been characterised: one with the highest specificity towards lysophosphatidic acid (lysoPthOH) and the other with the highest preference towards lysophosphatidylcholine (lysoPtdCho) [1, 3, 7, 8]. The former (Slc1) is encoded by the *SLC1* gene [1] and the latter by the *YOR175c* (other names: *ALE1, SCL4, LPT1*) gene [3, 8]. In the Arabidopsis genome, two genes (At1g12640 and At1g63050) encoding enzymes (AtLPCAT1 and AtLPCAT2) preferentially catalysing lysoPtdCho acyltransferase reaction have been found. Genes encoding enzymes with similar substrate specificity have been also cloned from *Ricinus communis*, *Hiptage benghalensis*, *Lesquerella fendleri* and *Carthamus tinctorius* [6]. Additionally, two Arabidopsis genes (At1g80950 and At2g45670) encoding LPLATs with preferences towards lysophosphatidylethanolamine (AtLPEAT1 and AtLPEAT2) have been characterised [9].

Phosphatidylcholine (PtdCho) plays a central role in plant lipid metabolism. It is the substrate for the production of polyunsaturated fatty acid outside the plastid [10]. PtdCho is also the substrate for the production of a number of unusual fatty acids such as hydroxy, epoxy, acetylenic and conjugated fatty acids, which occur in high amounts in seed triacylglycerols in some species [11]. The transfer of acyl groups formed on PtdCho to other lipids has been studied extensively and several mechanisms have been proposed [12–19]. In vitro studies suggested that plant LPCAT could operate reversibly producing lysoPtdCho and acyl-CoA from PtdCho and CoA [20]. After the plant LPCAT genes were cloned, it was shown that these enzymes, indeed, could operate reversibly in vitro [6]. Very recently, Pan et al. [22] delivered direct evidence that LPCAT can operate reversibly also in vivo. However, thus far, the existence of such an exchange mechanism of fatty acids has not been proven for other plant or yeast phospholipids.

In the presented study, we used in vitro assays to assess the abilities of two Arabidopsis and two yeast LPLAT enzymes to work in the forward and backward direction with different lysophospholipids (lysoPtdCho, lysoPtdEtn, lysoPtdOH) and phospholipids (PtdCho, PtdEtn, PtdOH) as acyl acceptors and acyl donors. The results are discussed in terms of the possible in vivo significance of the backward reactions in the re-modelling of the various phospholipids.

## Materials and Methods

### Chemicals

The non-radioactive fatty acids and acyl lipids were obtained from Laradon (Malmö, Sweden) and (1-^14^C)-labelled fatty acids were purchased from Perkin-Elmer Life Sciences. Bovine serum albumin (BSA) and free CoA were obtained from Sigma-Aldrich (St. Louis, MO, USA). The [1-^14^C]acyl-CoAs and acyl-CoAs were synthesised according to the modified method described by Sanchez et al. [23]. All the other chemicals were purchased from Sigma or Merck.

### Yeast Strains

The *Saccharomyces cerevisiae* haploid knock-out mutants of *ALE1* (BY4742; Matα; his3∆1; leu2∆0; lys2∆0; ura3∆0; YOR175c::kanMX4) were transformed either with the pYES2 plasmid (Invitrogen) carrying one of the four different gene-encoding enzymes with lysophospholipid:acyl-CoA acyltransferase activity (*At1g63050*, *At2g45670, YOR175c, SLC1*) under the control of a galactokinase 1 (GAL1) promoter, or with the empty pYES2 plasmid (control). The transformations were done according to the modified LiAc/SS carrier DNA/*PEG* method [24]. The yeasts were cultured on synthetic dropout medium lacking uracil and containing 2 % (w/v) galactose for the selection of transformants.

### Yeast Cultivation and Microsomal Preparation

The transformed yeast cells (either with empty plasmid or with one of the tested LPLAT genes) were cultured for 24 h on a rotating platform (220 rpm) at 30 °C in synthetic uracil drop-out medium containing 2 % glucose. After that time, galactose was added (2 % w/v final concentration) and the cells were grown for an additional 24 h. Microsomal fractions were prepared according to the method described in Dahlqvist et al. [25]. The yeast cultures (100 ml for a single preparation) were centrifuged and washed twice with distilled water. Each pellet was suspended in the 1 ml of ice-cold buffer containing 20-mM Tris/HCl, pH7.9, 10-mM MgCl_2_, 1-mM ethylenediaminetetraacetic acid (EDTA), 5 % (v/v) glycerol, 0.3-M ammonium sulphate and protease inhibitors (Complete, Roche Applied Sciences) and transferred to a 2-ml Eppendorf tube with 1-ml glass beads (0.45–0.5 mm in diameter). The tubes were shaken (10 times, 30 s) using the "Mini Bead Beater-8" (Biospec Products, Bartlesville, OK, USA) and the homogenates were centrifuged for 10 min at 1500×*g*. The resulting supernatants were transferred to the new tubes, and centrifuged at 100,000×*g* for 2 h. The pellets were suspended in the 0.1-M potassium phosphate buffer (pH7.2) and these extracts, subsequently referred to as microsomal fractions or microsomes, were stored at −80 ^°^C until they were used for further analyses. All the activities connected with the preparation of microsomal fractions were performed at 4 ^°^C.

### Enzyme Assays

Assays measuring activity and substrate specificity (towards different acyl acceptors) of the lysophospholipid:acyl-CoA acyltransferases in the forward reactions contained 10 nmol of [^14^C]18:1-CoA, 5 nmol *sn*-1-18:1-lyso-phospholipid (lysophosphatidylcholine—lysoPtdCho, lysophosphatidic acid—lysoPtdOH and lysophosphatidylethanolamine—lysoPtdEtn) with the amount of microsomes indicated in the tables or figures (optimized to achieved the maximal activity of the tested acyltransferases) in 100 µl of 40-mM potassium buffer (pH 7.2). The incubation time was 2 min at 30 ^°^C (with shaking at 1250 rpm).

In the presented study, we based our activity on the amount of endogenous PtdCho in the assays. We noted that the measurement of the content of endogenous PtdCho gives more accurate data than the measurements of the content of microsomal proteins. Since the enzymes assayed are all integral membrane proteins, this gives a better comparison of specific activities between the different microsomal preparations than calculated on microsomal proteins. To be able to compare our results with the results presented in other publications, we also measured the ratio of microsomal PtdCho/microsomal proteins, and in our laboratory, 1 nmol microsomal PtdCho corresponded approximately to 8 µg of microsomal proteins.

The assays measuring the backward reactions contained aliquots of microsomal fractions (5 nmol of endogenous PtdCho) together with 1 mg of BSA, 0.2 µmol of free CoA, 10 nmol of [^14^C]18:1-CoA in a total volume of 100 µl of 40-mM potassium buffer (pH 7.2). As background activity (i.e. incorporation of radioactivity into phospholipids through reactions other than the reverse reaction of LPLATs) 0.5 µmol dithionitrobenzoic acid (DTNB) was added to the reaction mixture [6]. The reactions were carried out in a "Thermomixer Compact" (Eppendorf) at 30 ^°^C with shaking (1250 rpm) for 2, 5, 10, 30 and 60 min. The amount of acyl group exchange was calculated by subtracting the radioactivity found in PtdCho, PtdEtn or PtdOH in the presence of DTNB (that inhibits the acyl exchange) from the radioactivity found in these lipids in assays in the absence of DTNB.

To validate the results obtained in the reverse reactions using the radioactive measurements, experiments with non-radioactive substrates and gas–liquid chromatography (GLC) analyses were performed. The assays were carried out similar to the described earlier assays of the backward reactions with radioactive acyl-CoA. The incubation time was 0, 30 and 60 min, and non-radioactive linoleoyl-CoA (18:2-CoA) was used instead of radioactive [^14^C]18:1-CoA. Since 18:2 is not present in yeast, the incorporation of this acyl group into the various phospholipids could be followed by GLC. The chloroform fractions from six assays were pooled together and used for lipids analyses. Two different microsomal fractions were analysed: control (membranes from yeast expressed with an empty vector) and from yeast overexpressing Arabidopsis LPCAT2.

Both the forward and the reverse reactions were terminated by the addition of 375 µl of chloroform/methanol (1:2; v/v), 5 µl of acetic acid, 125 µl of chloroform and 125 µl of water to the reaction mixture. After vortexing, the tubes were centrifuged (1000×*g*) for 2 min and the chloroform fractions (containing the lipids) were collected and used for further analyses.

### Lipid Separation and Analyses

Lipids extracted to the chloroform fractions were separated by thin layer chromatography (TLC) on silica gel 60 plates (Merck, New York, USA) using chloroform/methanol/acetic acid/water (85/15/10/3.5, v/v/v/v) as the solvent system. Radioactive lipids were visualised and quantified on the plate by electronic autoradiography (Instant Imager, Packard Instrument Co.). Non-radioactive lipids were visualised on the plate with I_2_ vapour and identified by means of standards. After I_2_ evaporation, gel from areas corresponding to the analysed lipid classes was removed and lipids were methylated in situ on the gel with 2 % H_2_SO_4_ in dry methanol (60 min at 90 ^°^C). The methyl esters were extracted with hexane and analysed by GLC equipped with a flame ionisation detector (FID) and 60 m × 0.25 mm CP-WAX 58 CB fused-silica column (Agilent Technologies, Santa Clara, CA, USA) with methyl-heptadeceanoic acid added as an internal standard.

## Results

### Lysophospholipid Specificities of LPLAT

**Fig. 1:**
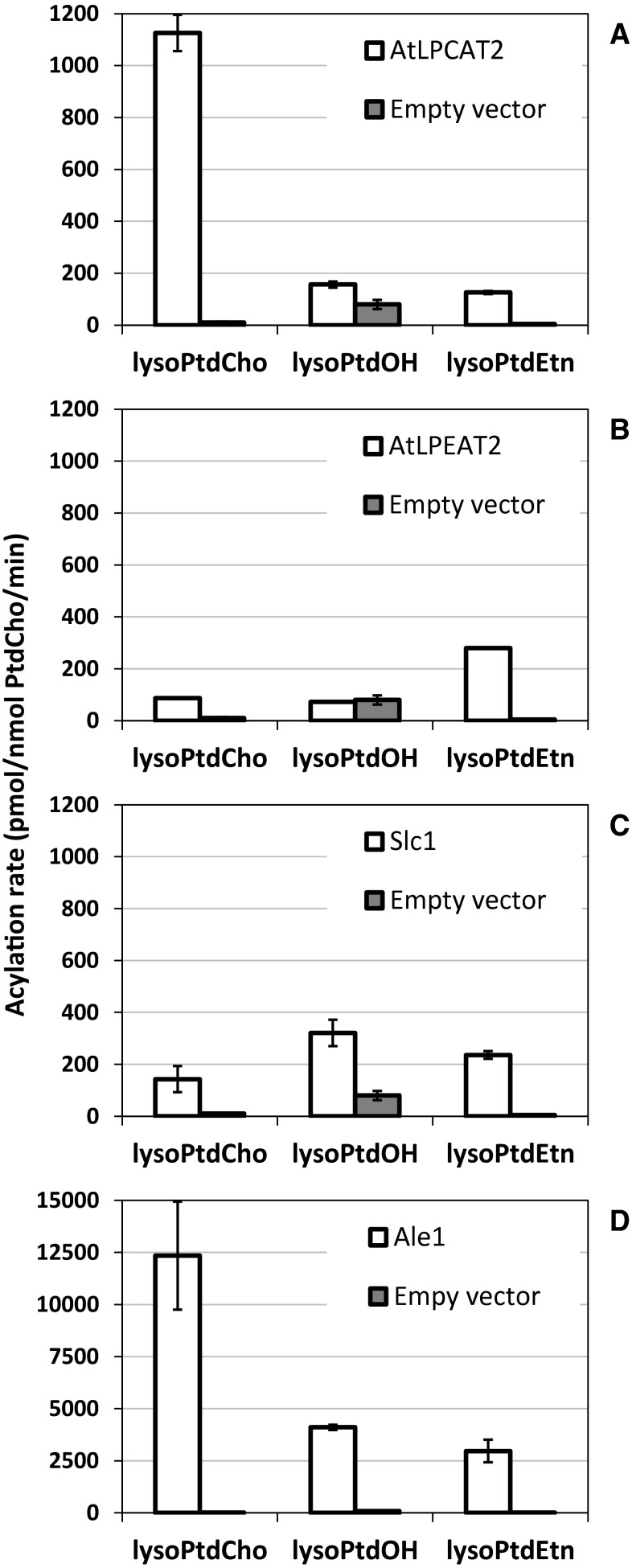
Activities of Arabidopsis and yeast LPLAT towards different lysophospholipids. *White bars*, transformed with LPLAT; *grey bar*, transformed with an empty vector. The assays contained microsomal fractions of yeast (*ale1*)-overexpressing genes encoding AtLPCAT2 (**a** 0.05 nmol of microsomal PtdCho), AtLPEAT2 (**b** 2 nmol of microsomal PtdCho), Slc1 (**c** 2 nmol of microsomal PtdCho) and Ale1 (**d**, 0.1 nmol of microsomal PtdCho), 10 nmol of oleoyl-lysoPtdCho, oleoyl-lysoPtdOH or oleoyl-lysoPtdEtn and 10 nmol of [^14^C]18:1-CoA in a total volume of 100 µl. Incubation was done at 30 ^°^C for 2 min. The data show are the mean ± standard deviation (SD) for triplicate samples

Two plant LPLATs, AtLPCAT2 and AtLPEAT2, and two yeast LPLATs, Slc1 and Ale1 were assayed using different oleoyl-lysophospholipid acyl acceptors in microsomal preparation of *ale1* knockout yeast strain expressing the different genes under the control of the GAL promotor (Fig. 1). The specificities obtained for AtLPCAT2 (Fig. 1a) were in good agreement with previous published results [3] with lysoPtdCho being by far the best substrate but also with some activity towards lysoPtdOH and lysoPtdEtn. In agreement with an earlier report [9], the best acyl acceptor for AtLPEAT2 was lysoPtdEtn and with some activity towards lysoPtdCho (Fig. 1b). We found no significant activities towards oleoyl-lysoPtdOH. This substrate was not tested by Stålberg et al. [9]. The lysophospholipid specificity of Slc1 was close to what was reported earlier [3], showing a broad acyl specificity by accepting well all lysophospholipids offered (Fig. 1c). Ale1 also showed broad acyl specificity, having high activities towards lysoPtdCho, lysoPtdOH, and lysoPtdEtn with highest activity towards lysoPtdCho (Fig. 1d). It has previously been shown that this enzyme can be used these acyl acceptors [7]. It should be noted that the specific activity of the Ale1 towards lysoPtdCho was about 10 times that of AtLPCAT2, which had the next highest specific activity of the tested LPLATs.

### Remodelling of Acyl Groups of PtdCho, PtdEtn and PtdOH Catalysed by the Backward and Forward Reactions of Different LPLATs

It was previously shown that backward reaction of AtLPCAT can be totally inhibited in microsomal preparations from both yeast and plants by adding DTNB [6, 26]. DTNB binds covalently to free CoA without affecting the forward reaction. The incorporation of acyl groups from acyl-CoA into the phospholipids without addition of lysophospholipids and in the presence of DTNB thus occurs by acylation of existing lysophospholipids in the membranes and lysophospholipids generated during the assay time by other means than the reversibility of LPLAT. The differences between incorporation of acyl groups from [^14^C]acyl-CoA in the absence and presence of DTNB in incubations in absence of added lysoPtdCho will thus be a measurement of the extent the phospholipid acyl groups incorporated via acyl exchange between the phospholipid and acyl-CoA.

**Fig. 2:**
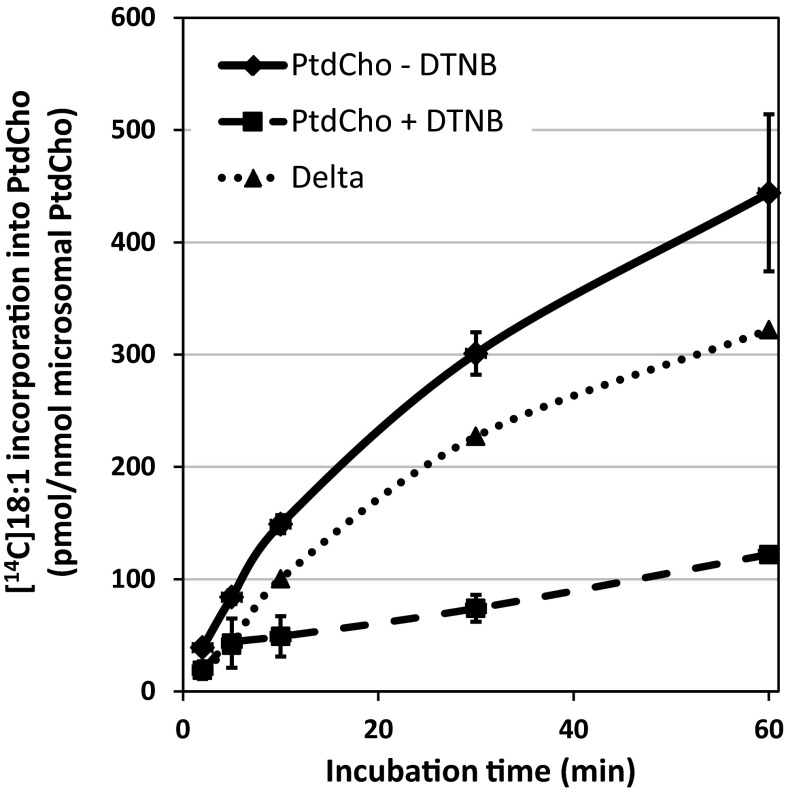
Time-course incorporation of [^14^C]acyl groups from [^14^C]18:1-CoA into PtdCho in microsomes prepared from *ale1* yeast expressing AtLPCAT2 in the presence and absence of DTNB. The delta graph depicts incorporation with DTNB subtracted from incorporation without DTNB and represent incorporation via acyl exchange (see Supplement Fig. 1). The values are the mean ± SD from quadruplicate samples

The differences in incorporation of [^14^C]18:1-CoA into PtdCho in microsomal preparations of *ale1* yeast expressing AtLPCAT2 with and without DTNB are shown in Fig. 2. An approximately 4-times higher amount was incorporated into PtdCho at 60 min in the absence of DTNB than in its presence. In Supplement Fig. 1, we use this experiment to describe how the incorporation via the combined reverse and forward reactions of the LPLAT is calculated.

**Fig. 3:**
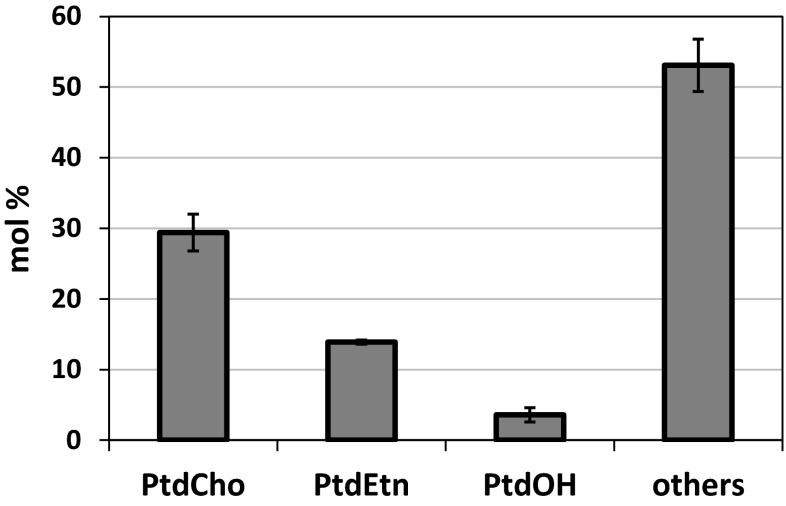
Relative content of phosphatidylcholine (PtdCho), phosphatidylethanolamine (PtdEtn), phosphatidic acid (PtdOH) and other acyl lipids in microsomal preparations of *ale1* yeast. The content of the different lipids is based on the assumption that all lipids have two acyl groups. The results are mean ± SD from triplicate samples

The degree of reversibility of a certain phospholipid substrate has to be judged on its relative abundance compared to other lipids in the microsomal membranes. We, therefore, determined the relative percentage of fatty acids in PtdCho, PtdEtn and PtdOH of all acyl lipids found in the microsomes (Fig. 3).

**Fig. 4:**
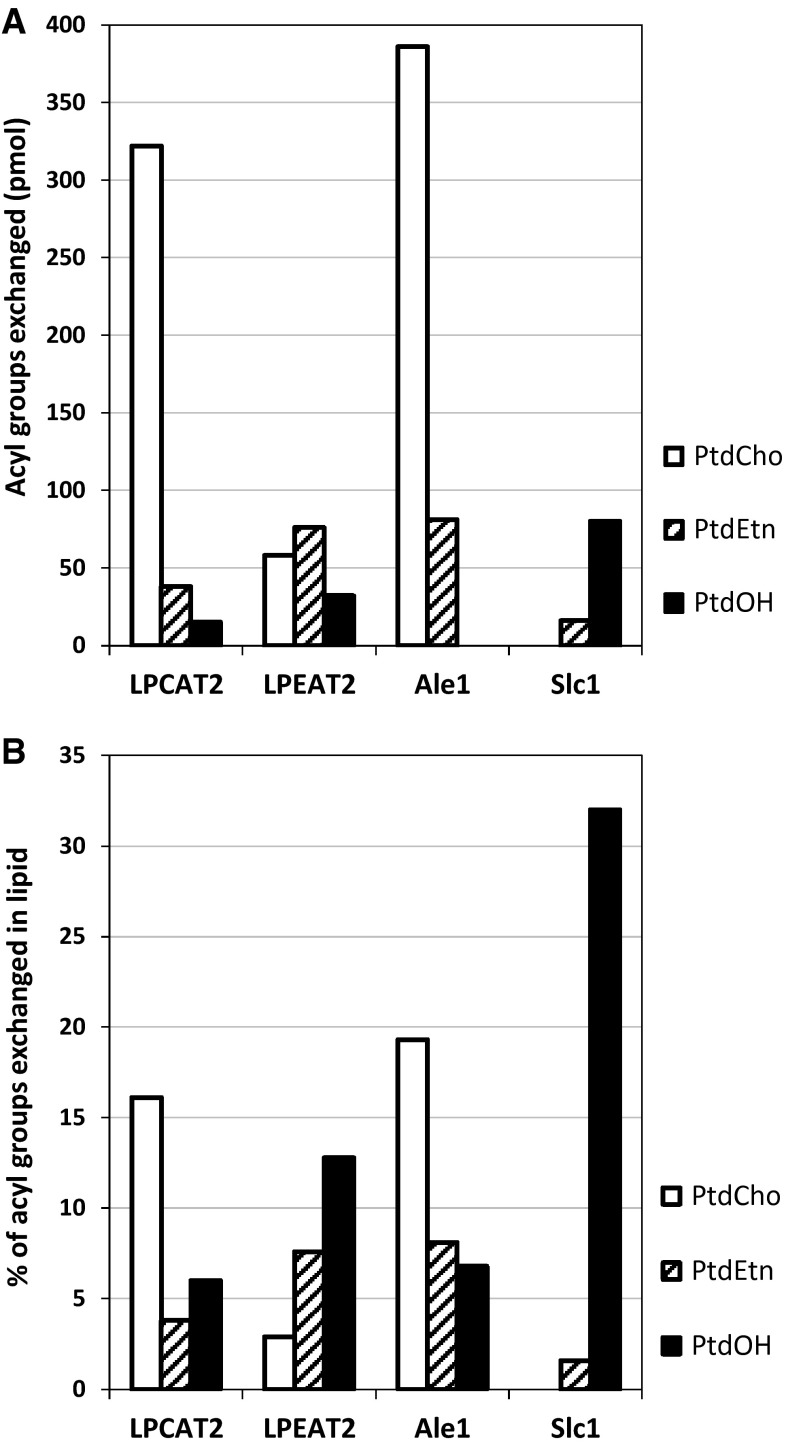
Incorporation of acyl groups from [^14^C]18:1-CoA into different phospholipids via acyl exchange catalysed by different LPLATs. **a** incorporation of [^14^C]18:1; **b** percentage of fatty acid exchanged in phospholipid. The data is from a 60-min incubation and depicts significant differences (mean difference via a two-sided test at *α* = 0.05) in incorporation in assays with and without DTNB (**a**) and calculated percentage of fatty acid exchanged based on the results presented in **a** and the lipid distribution presented in Fig. 3. Full data of the 60-min time-course with a mean of quadruplicate samples ± SD and statistical analysis are given in Supplement Tables S1, S2, S3, S4

We measured the extent of incorporation of [^14^C]18:1 from [^14^C]18:1-CoA into PtdCho, PtdEtn and PtdOH in the absence and presence of DTNB in microsomal preparations expressing AtLPCAT2, AtLPEAT2, Ale1 and Slc1. In Fig. 4, the differences in incorporation in the different phospholipids with and without DTNB at 60-min incubations are depicted. The Supplement Tables S1–S4 give the full results from time-course assays with a statistical evaluation. The amount of ^14^C-acyl groups incorporated via acyl exchange was highest and similar in PtdCho with AtLPCAT2 and Ale1 expressed, whereas AtLPEAT2 incorporated similar amounts in PtdCho and PtdEtn by exchange. No significant incorporation via acyl exchange occurred in PtdOH by Ale1 and in PtdCho by Slc1; despite that, they had high activity with the corresponding lyso substrates in the forward reaction (Fig. 1).

Since the amount of the different phospholipid differed considerably in the microsomal membranes (Fig. 3), we calculated the amount of acyl exchange shown in Fig. 4a as a percentage of acyl groups present in the respective phospholipid (Fig. 4b). Since the amount of PtdOH was very small in the membranes, the Slc1 exchanged over 30 % of the acyl groups in PtdOH in 60 min despite the low absolute amount of [^14^C]18:1 incorporated (compare Fig. 4a, b). AtLPCAT2 and Ale1 exchanged about 15 and 20 %, respectively, of the acyl groups in PtdCho during the same assay period. The extent acyl exchanged in PtdEtn was approximately similar in membranes expressing LPEAT2 and Ale1 and lower with AtLPCAT2 and Slc (Fig. 4b).

In order to confirm the correctness of these calculations, the same assay conditions were used with membranes from yeast expressing AtLPCAT2 but with adding non-radioactive 18:2-CoA instead of [^14^C]18:1-CoA. It has previously been shown that AtLPCAT2 has a similar exchange rate of PtdCho with 18:1-CoA and 18:2-CoA [6]. Since 18:2-CoA is not present in endogenous yeast lipids, we determined the relative amount of this acyl group in PtdCho and PtdEtn by GLC in the assays after various incubation periods. The data are presented in Supplement Table S5 and show good agreement with what is calculated in Fig. 4b on the basis of radioactive incorporation of [^14^C]18:1-CoA (Fig. 4a) and the relative distribution of the different phospholipids in the membranes (Fig. 3).

### The Relationship Between the Activities of the Forward and Reverse Reactions of LPLATs

**Fig. 5:**
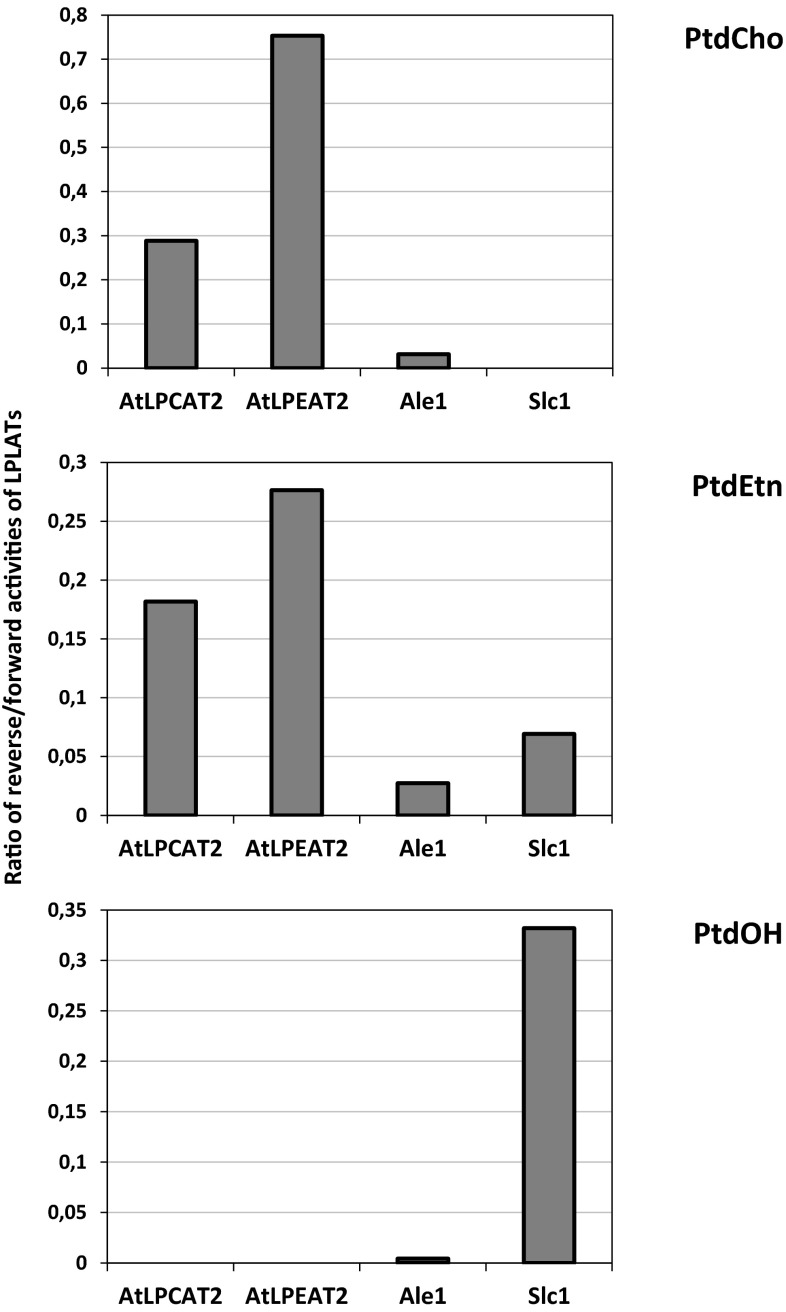
Ratio between reverse and forward activities for different LPLATs for different phospholipids. The data is given as ratios between amounts of radioactivity incorporated into lipids as presented in Figs. 4b and 1a

It should be noted that all the LPLAT enzymes assayed in our study were expressed with a strong GAL promotor and, thus, their specific activities in the membranes are likely to be much different than in their native membranes. Therefore, the significance of each enzyme's reversibility has to be judged by comparing the activity of the forward reactions with the backward reactions. We, therefore, calculated the ratio between the pmol (^14^C)-acyl groups incorporated by acyl exchange in each phospholipid after 60 min, as depicted in Fig. 4a, and the pmol of each phospholipid formed from lysophospholipids in the forward reaction after 2 min, as depicted in Fig. 1. Since the Slc1 gene was not deleted in the microsomes used and gave a high background in acylation of lysoPtdOH compared to the forward reactions in assays where AtLPCAT2 and AtLPEAT2 was expressed, it can also be assumed that a considerable part of the incorporation via acyl exchange into PtdOH with these two genes expressed could be a result of endogenous Slc1 expression. Therefore, PtdOH was excluded from these calculations when AtLPCAT2 and AtLPEAT2 were expressed. It should be noted that such a calculation does not represent any true ratio but only indicates the relative abilities of the different enzymes to operate backwards on the different phospholipids. Such a comparison of the ratios between the backward activities and forward reactions of the different LPLATs shows, surprisingly, that there is no correlation between the activity in the forward reaction and the backward reaction for the different enzymes (Fig. 5). Further, the ratio differed drastically between different phospholipids for a particular enzyme. Despite very high activity in the forward reaction by the Ale1, its ability to operate reversibly on all tested lipids is very low compared to AtLPCAT2 and AtLPEAT2. Slc1 has no detectable reverse reaction towards PtdCho substrate, but has the highest capacity of reversibility towards the PtdOH substrate of all tested enzymes.

## Discussion

The generally accepted mechanism in acyl remodelling of a phospholipid is the action of an A-type phospholipase of creating a lyosophospholipid and a free fatty acid and then re-acylating this lysophospholipid with acyl-CoA, the so called Land’s cycle [27]. An alternative mechanism was suggested by Stymne and Stobart [20] based on experiments with microsomal fraction of safflower seeds. In that work, it was suggested that LPCAT catalyses-remodelling of PtdCho was by an acyl exchange mechanism between the lipid and acyl-CoA where the removal of the acyl group from the phospholipid is carried out by the reverse reaction of the enzyme. After the plant LPCAT genes had been identified, Lager et al. [6] confirmed that plant LPCATs can catalyse acyl exchange in vitro in microsomal preparations. The most compelling indication that this was also occurring in vivo was the discovery that AtLPCAT had high selectivity for the removal of ricinoleoyl (12-hydroxy-octadece-9-enoyl) groups from PtdCho [6]. Bates and Browse [28] showed that in developing seeds of Arabidopsis transformed with castor bean hydroxylase, which use oleoyl-PtdCho as a substrate, 50 % of their newly synthesised diacylglycerols had a ricinoleoyl group, whereas the PtdCho had only 2 % of this acid. This indicated a high selectivity for the transfer of ricinoleoyl groups over other acyl groups from PtdCho into the acyl-CoA pool in Arabidopsis, in line with what was found in the in vitro assays of the reverse reaction of AtLPCAT2 [6]. Very recently, Pan et al. [22] delivered direct evidence that LPCAT can operate reversibly in vivo. They showed that when an *ale1* yeast strain deficient in storage lipid synthesis and expressing Δ12 and Δ15 PtdCho desaturases (FAD2 and FAD3) was transformed with a diacylglycerol acyltransferase in combination with a linseed LPCAT, the level of polyunsaturated fatty acid in triacylglycerols (TAG) more than doubled and the amount in phospholipids decreased by 40 % compared to if only the DGAT was expressed. Since plant LPCATs do not possess phospholipase activity [6], the only reasonable explanation for this increase in TAG and decrease in phospholipids is that the plant LPCATs, by its backward reaction, transferred the polyunsaturated acyl groups formed on phospholipids into the acyl-CoA pool, which then were used by the DGAT to synthesise TAG.

In this paper, we investigated the ability of different LPLATs to catalyse the forward and reverse reactions for three different phospholipids, PtdCho, PtdEtn and PtdOH. Although all the LPLATs could catalyse the reverse reaction, the activity of the reverse reaction varied greatly between phospholipids and did not correlate with the rate of the forward activity. The conditions that would promote the reverse reaction of LPLATs in vivo have been discussed in some details by Lager et al. [6] and Pan et al. [22] and include low levels of lysophospholipids, high amounts of phospholipid substrate and low concentrations of available acyl-CoAs. However, the work presented here show, rather surprisingly, that some of the LPLATs have developed properties that, to a much higher degree than other LPLATs, promote the reverse reaction during the same assay conditions and with the same phospholipid. The data also show that the capacity of reversibility can be specific for a particular phospholipid, albeit the lysophospholipid derivatives of other phospholipids serve as good acyl acceptors for the forward reaction of the enzyme. It has previously been shown that ricinoleoyl groups are very poor acyl donors for AtLPCAT in the forward reaction, whereas they are far better acyl donors in the reverse reaction [6]. Since the acyl groups are attached to a soluble molecule in the forward reaction and to a membrane lipid in the reverse, it can be anticipated that the presentation of the acyl group to the enzyme will be very different in the two reactions and this could explain the different acyl selectivity between the forward and reverse reactions. AtLPCAT2 and ALE1 are membrane bound O-acyl transferase (MBOAT) proteins, whereas LPEAT and SLC1 are PlsB types of acyltransferases and these two protein families are very different in structure and this will probably also be reflected in different endoplasmic reticulum (ER) localization profiles which might contribute to their abilities to work reversibly.

Since the genes encoding the different LPLATs are driven by a strong GAL promotor in our yeast experiments, it is likely that the gene expressions are quite different than in cells expressing the genes under their natural promotor. Further, we do not know how well these genes are translated to proteins in the yeast cells and how fast these proteins are degraded. It is, therefore, difficult to predict the in vivo importance of the different LPLATs in remodelling the different phospholipids by their reverse reaction from these experiments. If we compare the ability of the two enzymes that have high activity in acylation of lysoPtdCho, i.e. AtLPCAT2 and Ale1 to operate reverse, we can conclude that the degree of reversibility of Ale1 is only one tenth of AtLPCAT2. It has previously been shown [6] that LPCAT1 has about the same rate of the reversible reaction as AtLPCAT2 despite that its forward activity is only about 20–30 % of the AtLPCAT2 activity. Thus, our data indicate that if AtLPCATs and Ale1 have similar specific activity in the forward reactions in a membrane, the acyl exchange rate catalysed by Ale1 will be only one tenth of that of the AtLPCAT. The only substrate used to any significant extent in the reverse reaction by Slc1 was PtdOH and, to a lower extent, PtdEtn; despite that, lysoPtdCho was also a good substrate in the forward reaction . It is interesting to note that a lysophosphatidic acid acyltransferase (LPAAT) from a mouse has been reported to also act reversibly with PtdOH as the substrate [21]. It should be noted that the amount of PtdOH in the membranes in an intact cell is very low and rapidly turned over by conversion to DAG by the phosphatidic acid phosphatases. It is, therefore, not likely that Slc1 or any other acylating enzyme producing PtdOH will act efficiently in reverse in vivo. LPEAT2 had the highest ratio of reversible to forward reactions with PtdEtn of all the enzymes tested (Fig. 5) and also had higher activity than AtLPCAT2 in acylating lysoPtdEtn (Fig. 1). It can be hypothesised that this enzyme plays as important role in acyl remodelling of PtdEtn by acyl exchange as AtLPCAT does with PtdCho.

It is the combined forward and reverse reactions of an enzyme that catalyse an exchange of acyl groups between a phospholipid pool and acyl-CoA pool where the lysophospholipids formed by the backward reaction are immediately re-acylated in the forward reaction. In absence of an acyl-CoA pool to mix with, the acyl-CoA formed in reverse reaction will be immediately re-acylated with the formed lysophospholipids and, thus, the reverse reaction will be impossible to observe. As Pan et al. [22] have pointed out, the driving force for the channelling of a particular acyl group from a phospholipid to other lipids by acyl exchange is, thus, the utilisation of that particular acyl group by other acyl-CoA acyl transferases, such as the acylation enzymes of the glycerol 3-phosphate pathway.

In conclusion, in the absence of good phospholipase A candidates for observed acyl remodelling of phospholipids through Land’s pathway [27], remodelling through the reverse reaction of LPLATs should be considered, not only in plants but also in other phyla. The work presented here and the work of Lager et al. [6] show that such remodelling can be both quite acyl- and lipid-specific and cannot be deduced from the specific activity of the forward reaction of the LPLAT.

## Electronic supplementary material

Supplementary material 1 (DOCX 141 kb)

## Abbreviations

PtdCho: Phosphatidylcholine
lysoPtdCho: Lysophosphatidylcholine
PtdOH: Phosphatidic acid
lysoPtdOH: Lysophosphatidic acid
PtdEtn: Phosphatidylethanolamine
lysoPtdEtn: Lysophosphatidylethanolamine
TAG: Triacylglycerol
DAG: Diacylglycerol
18:1: 18:1n-9, oleic acid
18:2: 18:2n-6, linoleic acid
LPLAT: Lysophospholipid:acyl-CoA acyltransferase
LPAAT: Lysophosphatidic acid:acyl-CoA acyltransferase
LPCAT: Lysophosphatidylcholine:acyl-CoA acyltransferase
LPEAT: Lysophosphatidylethanolamine:acyl-CoA acyltransferase
DGAT: Acyl-CoA:diacylglycerol acyltransferase
