# Supplementary material for: Possible Role of Different Yeast and Plant Lysophospholipid:Acyl-CoA Acyltransferases (LPLATs) in Acyl Remodelling of Phospholipids
Source: Lipids. 2015 Dec 7;51:15–23. doi: 10.1007/s11745-015-4102-0 (PMC4700060; doi:10.1007/s11745-015-4102-0)
Supplement: Supplementary file 1 — Supplementary material 1 (DOCX 141 kb) [file 11745_2015_4102_MOESM1_ESM.docx]

**Supplementary materials**

**Possible role of different yeast and plant lysophospholipid:acyl-CoA acyltransferases (LPLAT) in acyl remodelling of phospholipids**

**LIPIDS (2015)**

Katarzyna Jasieniecka-Gazarkiewicz^1^, Kamil Demski^1^, Ida Lager^2^,

Sten Stymne^2^, Antoni Banaś^1*^

^1^Intercollegiate Faculty of Biotechnology of University of Gdańsk and Medical University of Gdańsk, Kładki 24, 80-822 Gdańsk, Poland

^2^Department of Plant Breeding, Swedish University of Agricultural Sciences, 230 53 Alnarp, Sweden

* Corresponding author. Tel.: +48 58 523 63 44

*E-mail address*: [banas@biotech.ug.gda.pl](mailto:banas@biotech.ug.gda.pl) (A. Banaś).

**Table S1.** Incorporation of [^14^C]18:1 (added to the reaction mixture as [^14^C]18:1-CoA) into different polar lipids of microsomal fraction of yeast overexpressed with Arabidopsis **LPCAT2** in incubations without and with addition of DTNB.

| Incubation  time  [min] | pmol/1nmol microsomal PtdCho | | | | | | | | |
| --- | --- | --- | --- | --- | --- | --- | --- | --- | --- |
|  | PtdCho | | | PtdEtn | | | PtdOH | | |
|  | -DTNB | +DTNB | ∆ | -DTNB | +DTNB | ∆ | -DTNB | +DTNB | ∆ |
| 2 | **39**  ±4 | **19^a^**  ±8 | **20**  (1) | **2.7**  ±0.9 | **3.1**  ±0.5 | **-0.4** | **2.6**  ±0.5 | **2.3**  ±0.3 | **0.3** |
| 5 | **84**  ±6 | **43^a^**  ±22 | **41**  (2.1) | **6.9**  ±1.1 | **7.8**  ±1.5 | **-0.9** | **7.1**  ±1.6 | **5.4**  ±0.8 | **1.7** |
| 10 | **149**  ±8 | **49^a^**  ±18 | **100**  (5) | **15**  ±2 | **12^a^**  ±1 | **3**  (0.3) | **18**  ±4 | **10^a^**  ±2 | **8**  (3.2) |
| 30 | **301**  ±19 | **74^a^**  ±12 | **227**  (11.3) | **32**  ±8 | **19^a^**  ±2 | **13**  (1.3) | **29**  ±6 | **15^a^**  ±2 | **14**  (5.6) |
| 60 | **444**  ±70 | **122^a^**  ±6 | **322**  (16.1) | **55**  ±15 | **22^a^**  ±2 | **33**  (3.3) | **35**  ±3 | **20^a^**  ±1 | **15**  (6) |

∆ = (-DTNB) – (+DTNB); In bracket is calculated % of endogenous phospholipid’s fatty acids exchanged for [^14^C]18:1 from [^14^C]18:1-CoA pool The results presented are mean ±S.D. for at least quadruplicate assays.

^a^ = significant difference between (-DTNB) and (+DTNB) in mean difference two-sided test at α = 0.05

**Table S2.** Incorporation of [^14^C]18:1 (added to the reaction mixture as [^14^C]18:1-CoA) into different polar lipids of microsomal fraction of yeast overexpressed with Arabidopsis **LPEAT2** in incubations without and with addition of DTNB.

| Incubation  time  [min] | pmol/1nmol microsomal PtdCho | | | | | | | | |
| --- | --- | --- | --- | --- | --- | --- | --- | --- | --- |
|  | PtdCho | | | PtdEtn | | | PtdOH | | |
|  | -DTNB | +DTNB | ∆ | -DTNB | +DTNB | ∆ | -DTNB | +DTNB | ∆ |
| 2 | **15**  ±1 | **12**  ±2 | **3** | **33**  ±6 | **29**  ±11 | **4** | **18**  ±2 | **17**  ±3 | **1** |
| 5 | **21**  ±1 | **26**  ±6 | **-5** | **55**  ±16 | **51**  ±21 | **4** | **42**  ±6 | **33**  ±5 | **9** |
| 10 | **32**  ±5 | **36**  ±5 | **-4** | **85**  ±5 | **77**  ±7 | **8**  (0.8) | **60**  ±7 | **57**  ±16 | **3** |
| 30 | **77**  ±13 | **58**  ±17 | **19**  (1) | **155**  ±12 | **117^a^**  ±12 | **38**  (3.8) | **83**  ±8 | **66**  ±12 | **17**  (6.8) |
| 60 | **157**  ±6 | **99^a^**  ±5 | **58**  (2.9) | **176**  ±31 | **107^a^**  ±18 | **69**  (6.9) | **119**  ±19 | **87^a^**  ±8 | **32**  (12.8) |

∆ = (-DTNB) – (+DTNB); In bracket is calculated % of endogenous phospholipid’s fatty acids exchanged for [^14^C]18:1 from [^14^C]18:1-CoA pool The results presented are mean ±S.D. for at least quadruplicate assays.

^a^ = significant difference between (-DTNB) and (+DTNB) in mean difference two-sided test at α = 0.05

Table S3. Incorporation of [14C]18:1 (added to the reaction mixture as [^14^C]18:1-CoA) to different polar lipids of microsomal fraction of yeast overexpressed with **Ale1** during incubation without and with addition of DTNB in assays favouring backward reactions.

| Incubation  time  [min] | pmol/1nmol microsomal PtdCho | | | | | | | | |
| --- | --- | --- | --- | --- | --- | --- | --- | --- | --- |
|  | PtdCho | | | PtdEtn | | | PtdOH | | |
|  | -DTNB | +DTNB | ∆ | -DTNB | +DTNB | ∆ | -DTNB | +DTNB | ∆ |
| 2 | **118**  ±22 | **64**  ±39 | **54**  (2.7) | **28**  ±10 | **16**  ±4 | **12**  (1.2) | **13**  ±7 | **17**  ±3 | **-4** |
| 5 | **206**  ±20 | **70^a^**  ±38 | **136**  (6.8) | **54**  ±19 | **24^a^**  ±5 | **30**  (3) | **33**  ±20 | **32**  ±8 | **1** |
| 10 | **245**  ±32 | **74^a^**  ±18 | **171**  (8.6) | **72**  ±28 | **27^a^**  ±9 | **45**  (4.5) | **43**  ±7 | **40**  ±9 | **3**  (1.2) |
| 30 | **343**  ±83 | **139^a^**  ±10 | **204**  (10.2) | **104**  ±38 | **47^a^**  ±11 | **57**  (5.7) | **55**  ±9 | **45**  ±11 | **10**  (4) |
| 60 | **546**  ±89 | **160^a^**  ±16 | **386**  (19.3) | **136**  ±17 | **55^a^**  ±11 | **81**  (8.1) | **88**  ±20 | **71**  ±12 | **17**  (6.8) |

∆ = (-DTNB) – (+DTNB); In bracket is calculated % of endogenous phospholipid’s fatty acids exchanged for [^14^C]18:1 from [^14^C]18:1-CoA pool The results presented are mean ±S.D. for at least quadruplicate assays.

^a^ = significant difference between (-DTNB) and (+DTNB) in mean difference two-sided test at α = 0.05

**Table S4.** Incorporation of [^14^C]18:1 (added to the reaction mixture as [^14^C]18:1-CoA) to different polar lipids of microsomal fraction of yeast overexpressed with **Slc1** during incubation without and with addition of DTNB in assays favouring backward reactions.

| Incubation  time  [min] | pmol/1nmol microsomal PtdCho | | | | | | | | |
| --- | --- | --- | --- | --- | --- | --- | --- | --- | --- |
|  | PtdCho | | | PtdEtn | | | PtdOH | | |
|  | -DTNB | +DTNB | ∆ | -DTNB | +DTNB | ∆ | -DTNB | +DTNB | ∆ |
| 2 | **2.1**  ±0.2 | **3.0**  ±0.7 | **-0.9** | **5.0**  ±1.1 | **5.2**  ±1.3 | **-0.2** | **13**  ±0.4 | **10.3^a^**  ±0.8 | **2.7**  (1.1) |
| 5 | **9.8**  ±0.6 | **11**  ±1 | **-1.2** | **11.9**  ±1.2 | **7.1^a^**  ±1.6 | **4.8**  (0.5) | **25**  ±3 | **22**  ±2 | **3**  (1.2) |
| 10 | **21**  ±3 | **20**  ±2 | **1** | **17**  ±1 | **10^a^**  ±1 | **7**  (0.7) | **56**  ±3 | **43^a^**  ±4 | **13**  (5.2) |
| 30 | **46**  ±3 | **52**  ±2 | **-6** | **26**  ±2 | **14^a^**  ±1 | **12**  (1.2) | **107**  ±3 | **69^a^**  ±5 | **38**  (15.2) |
| 60 | **74**  ±2 | **78**  ±5 | **-4** | **35**  ±3 | **19^a^**  ±1 | **16**  (1.6) | **173**  ±8 | **93^a^**  ±12 | **80**  (32) |

∆ = (-DTNB) – (+DTNB); In bracket is calculated % of endogenous phospholipid’s fatty acids exchanged for [^14^C]18:1 from [^14^C]18:1-CoA pool. The results presented are mean ±S.D. for at least quadruplicate assays.

^a^ = significant difference between (-DTNB) and (+DTNB) in mean difference two-sided test at α = 0.05

Table S5. Changes in fatty acid composition of PtdCho and PtdEtn of microsomal fraction of control yeast and yeast overexpressed with LPCAT2 during incubation with 18:2-CoA as determined by GLC.

| Microsomal fraction | Lipid | Time of incubation [min] | FA [mol%] | | | | |
| --- | --- | --- | --- | --- | --- | --- | --- |
|  |  |  | 16:0 | 16:1 | 18:0 | 18:1 | 18:2 |
| pYES2 | PtdCho | 0 | 24.9 | 34.4 | 6.9 | 33.8 | **0.0** |
|  |  | 30 | 25.0 | 34.4 | 7.0 | 32.9 | **0.7** |
|  |  | 60 | 24.8 | 34.7 | 7.0 | 32.7 | **0.8** |
|  | PtdEtn | 0 | 23.0 | 35.1 | 1.6 | 40.3 | **0.0** |
|  |  | 30 | 23.4 | 35.4 | 1.9 | 39.0 | **0.3** |
|  |  | 60 | 23.7 | 35.7 | 1.7 | 38.0 | **0.9** |
| LPCAT2 | PtdCho | 0 | 17.4 | 41.8 | 6.0 | 34.8 | **0.0** |
|  |  | 30 | 16.4 | 35.4 | 6.1 | 32.4 | **9.7** |
|  |  | 60 | 16.2 | 32.9 | 6.3 | 31.9 | **12.7** |
|  | PtdEtn | 0 | 12.1 | 39.3 | 1.5 | 47.1 | **0.0** |
|  |  | 30 | 12.1 | 39.6 | 1.0 | 45.7 | **1.8** |
|  |  | 60 | 12.2 | 38.4 | 1.1 | 45.4 | **2.9** |

Assays condition/tube: microsomal fraction (5nmol endogenous PtdCho), BSA (1mg/assay), CoA (0.2 µmol /assay), 18:2-CoA (10nmol), 100 µl 40 mM p-buffer (pH 7.2); incubation at 30^o^C with shacking (1250 rpm); B&D + 3 x additional extraction with 250 µl of chloroform; chloroform fraction from 6 assays polled together and separated on TLC (chloroform:methanol:acetic acid:water; 85:15:10:3.5; v:v:v:v); PtdCho and PtdEtn for GC analyses.

**Supplement Figure 1. Measurement of the reverse reaction of AtLPCAT2.**

Microsomes expressing AtLPCAT2 continuously incorporate acyl groups from acyl-CoA into PtdCho in absence of added lysoPtdCho (reaction A & B). The intensity of this reaction depended on the level of endogenous and formed *de novo* lysoPtdCho. In reaction A except endogenous one, lysoPtdCho is generated *via* LPCAT reverse reaction and *via* other reactions, e.g. phospholipases, PDAT. By adding DTNB, all free CoA is bound (reaction B) and the reverse reaction of the LPCAT cannot take place. Incorporation of acyl groups from acyl-CoA into PtdCho is *via* acylation of endogenous lysoPtdCho in the microsomes and lysoPtdCho generated during the incubation by other than LPCAT reverse reaction, e.g. by phospholipases, PDAT. The differences between reaction A and B will give the amount of acyl groups incorporated from added acyl-CoA *via* the combined reverse and forward reaction of the LPCAT.

lysoPtdCho * - endogenous and generated *via* other than LPCAT reverse reaction, e.g. phospholipases, PDAT

lysoPtdCho ** -generated *via* LPCAT reverse reaction, endogenous one and generated *via* other reaction, e.g. phospholipases, PDAT
